# An evaluation of Scottish green health prescriptions using the APEASE criteria

**DOI:** 10.1186/s12875-025-02746-9

**Published:** 2025-02-22

**Authors:** Neil Howlett, Imogen Freethy, Sian Harding, Adam P. Wagner, Lisa Miners, Honey Anne-Greco, Laura Lamming, Nigel Lloyd, Katherine E. Brown

**Affiliations:** 1 Public Health and Applied Behaviour change laboratory (PHAB Lab), Department of Psychology, Sport, and Geography, University of Hertfordshire, College Lane, Hatfield, AL10 9AB UK; 2 Norwich Medical School, University of East Anglia, Norwich Research Park, Norwich, NR4 7TJ UK; 3 National Institute for Health and Care Research (NIHR) Applied Research Collaboration (ARC) East of England (EoE), Cambridge, UK

**Keywords:** Green social prescribing, Green health activity, Green health partnerships, Green health prescriptions, APEASE

## Abstract

**Background:**

Time spent in green space such as parks and forests can have positive effects on physical and mental health. Green Health Partnerships were set up in Scotland to promote use of green space for health improvement. One of the main mechanisms to achieve this was the setup of Green Health Prescriptions (GHPr). This study evaluates three GHPrs in different localities across a range of feasibility elements, and the funding and resourcing associated with implementation.

**Methods:**

Interviews were conducted across service user, referrer, link worker, and activity provider groups across Dundee, Highland, and North Ayrshire. Interviews were deductively analysed using the APEASE (Acceptability, Practicability, Effectiveness, Affordability, Spillover effects, Equity) criteria. Data within each APEASE domain was then inductively coded producing more reflexive sub-themes. Data on funding and resources associated with delivering each programme was also collected to provide further context to the APEASE criteria.

**Results:**

All stakeholder groups generally found the concept of using green spaces and the GHPr acceptable, and, although service users perceived that staff were often good communicators, there were times where awareness of and knowledge about the GHPr were lacking. There were reported improvements across a wide range of physical and mental health, and social outcomes for service users. The GHPr was also considered affordable in terms of the green health activity sessions. A key issue for staff across practicability, acceptability, and with monitoring equity, was the lack of underpinning IT infrastructure for referrals, communication with link workers, and data capture to reflect on service user progress. As implemented in Dundee, progression through the GHPr, after initial referral, took on average 195 min, at a cost of £64 per service user.

**Conclusions:**

This evaluation highlighted the potential benefits for service users that can be realised through a GHPr. However, a lack of supportive systems to capture referral information, communicate between professionals, and document service user progress limits a more robust and extensive evaluation of the current GHPr model.

**Evaluation registration:**

Research Registry identifier: researchregistry9069, registration date: 25/04/23.

**Supplementary Information:**

The online version contains supplementary material available at 10.1186/s12875-025-02746-9.

Latest data show that in Scotland, population mental health and wellbeing levels are declining, and are lowest amongst those living in the most deprived areas [1]. Around two-thirds of Scottish adults are living with overweight or obesity, with higher prevalences among men, and people living in the most deprived areas. Only 65% of adults report meeting the physical activity guidelines, and reported sitting time per day has increased since 2015 [1]. Using a pre-populated list of options, the most common reason reported by Scottish Health survey respondents for not meeting physical activity guidelines was that their health was not good enough [1]. This reflects that more than one-third of Scottish adults are experiencing chronic pain, with a higher prevalence among women, and points to a population in need of preventative efforts to help them maintain or improve their physical and mental health.

## Green spaces and green health programmes

There is an extensive literature exploring the link between use of ‘green space’ areas (e.g., parks, forests, or community gardens) in the local environment and a range of physical and mental health outcomes. Green space can have beneficial effects on mental health such as reductions in stress and anxiety, and improvements in mood [2]. There may also be a dose-response effect with greater duration and frequency of visits linked to greater benefits [3]. Additionally, there are a range of physical health outcomes that can be impacted, many of which are related to long-term conditions. Green space exposure is associated with decreased salivary cortisol (stress hormone levels), heart rate, blood pressure, cholesterol, and decreased risk of preterm birth, small size for gestational age, type II diabetes, cardiovascular mortality, and all-cause mortality [4].

The health behaviour that may have the most synergistic effects with exposure to green spaces is physical activity, with evidence suggesting that physical activity programmes promoting the use of green space can increase use of, and physical activity in, urban green spaces [5]. Physical activity is also one of the mechanisms by which green spaces can affect health outcomes. People who visit green spaces for greater durations may experience lower rates of depression and blood pressure, and those who visit more frequently can have improved perceptions of social cohesion in their community [3]. Other potential mechanisms by which health is positively impacted by green space include reducing harm (e.g., exposure to air pollution), improving attention and physiological stress recovery, and through facilitating social cohesion in communities [6]. Limited local green space was also associated with feelings of loneliness and a lack of social support, which affected the relationship between green space and health [7]. Overall, exposure to green space shows a range of health benefits, albeit the findings are limited by the lack of causal evidence. Green space exposure is often beneficial when both quantity and quality are increased and when it affects health through increased social contact and nature-based physical activity.

## Green health partnerships

Green Health Partnerships in Dundee, North Ayrshire, and Highland were set up in 2018 to encourage the health, social care, environment, leisure, sport and active travel sectors to collaborate in making better use of local green space as a health-promoting resource [8]. Monitoring and evaluation of the partnerships from June 2018 to September 2021 showed that these partnerships achieved a range of objectives including: facilitating or promoting opportunities for green health activities; undertaking awareness raising and capacity building activities; establishing or facilitating referral pathways; coordinating and delivering outreach and information activities; and inclusion of green health in local policies and plans [9]. One of the aims of Green Health Partnerships is the setup and implementation of Green Health Prescriptions (GHPr), where service users receive a ‘prescription’ from a healthcare professional, community service, or self-refer. Early scoping work with a range of professionals found the perception that GHPrs would be a good strategic fit with public health priorities [10]. Further work with NHS healthcare professionals, service-users, and delivery partners in Dundee, showed support for the GHPr prior to its inception [11]. One of the key early recommendations was that GHPrs should include strong routine data collection to provide evidence of success [10]. Additional important needs identified included: a sustainable funding model; a centralised hub for all information and guidance related to the pathway; and that green health activities should be tailored to individual needs [11].

## Pragmatic evaluations and APEASE

A pragmatic evaluation approach was chosen because it attempts to identify ‘what works’, rather than seeking objective ‘truth’ or ‘reality’ [12]. Experimental designs such as randomised controlled trials (RCT) are often considered the optimal scientific method, particularly when assessing the efficacy/effectiveness of interventions. However, RCTs are routinely conducted with resources and within settings that cannot be replicated outside of a trial context [13]. Pragmatic evaluations are consistent with a socio-ecological approach to public health research [14], where the social, political, environmental, and cultural context of individuals or groups is considered crucial [15]. Aims of a pragmatic evaluation are to maximize the applicability of evaluation findings to real world service delivery [16], and to generate evidence that is contextually relevant, and therefore, of value to partners and wider stakeholders. This study explored the Acceptability, Practicability, Effectiveness, Affordability, Equity, and Spillover effects of the GHPrs (APEASE criteria; [17–18]). The APEASE framework is a systematic method to explore the acceptability and feasibility of a programme during implementation (e.g., [19]).

## Current study

Exposure to green space has positive benefits for physical and mental health, and therefore, programmes that prescribe tailored green health activities to individual service users could support both health promotion and act as low-intensity contribution to treatment for a range of conditions. With continuing pressure on public health budgets, programmes that use existing community assets have the potential to be important preventative tools. Despite the potential of GHPrs to achieve benefits, perspectives from existing referrers, link workers, service providers, and service users on what can influence implementation have yet to be explored. The current study focused on evaluating the implementation of GHPrs in Dundee, North Ayrshire, and Highland, and aimed to provide recommendations for evaluation partners and future potential funders, by speaking to a range of people involved in organising and running the GHPrs. This study provides a unique contribution as the first study to evaluate GHPrs using the APEASE framework to highlight how and why the programmes worked (or not), and the benefits service users might have had as a result. This research aimed to answer the following question:

- Are GHPrs in Dundee, Highland, and North Ayrshire considered to be acceptable, practicable, effective, affordable, beneficial to people or groups equally, are there any negative side-effects, and what are the costs and resources involved?

## Method

### Evaluation registration

This evaluation was pre-registered on Research Registry (Registry identifier: researchregistry9069).

### Green health partnerships

Green Health Partnerships were set up in Dundee, North Ayrshire, and Highland as part of the ‘Our Natural Health Service programme’, led by NatureScot [8]. Set up in 2018, their aim was to promote use of green space for health improvement. One of the major objectives was to establish and implement GHPrs, where service users receive a ‘prescription’ from a healthcare professional or community service (e.g., a GP, pharmacist, or other Allied Health Professionals). This is more closely related to a care plan recommendation (which is responsive to service user needs and preferences) than a medical prescription. The GHPr pathway involves a referral, a consultation (by phone or in person) with a link worker or green health coordinator, matching with an appropriate green health activity, provision of information about the group, and then attendance at a green health activity most suitable for the service user. In some areas there was also the option to self-refer. The GHPr allows healthcare professionals to connect service users with free or low-cost outdoor activities delivered and supported by the third sector, including charities and volunteer groups. Differences in setup and geography across the three areas, at the time of the evaluation, are expanded upon below.

### Dundee

Dundee is the fourth largest city in Scotland with a population of approximately 150,000, with 41% of urban areas comprised of publicly accessible greenspace, albeit this equates to only 16 hectares of green space per 1,000 people [20]. Dundee had a structured GHPr pathway with allocated funding and a dedicated Green Health coordinator, website, and data management system. Service users could be referred to a green health activity via any NHS primary (e.g., GP) or secondary healthcare (e.g., physiotherapist) professional. Local people were supported to access and engage with over 60 outdoor opportunities across the city. The Dundee Green Health Partnership also worked to improve engagement with nature for specific target groups, such as NHS inpatients.

### Highland

Highland covers one third of the land mass of Scotland, with a population of approximately 235,000 people, and includes the most remote parts of the United Kingdom, with 47% of urban areas comprised of publicly accessible greenspace and 63 hectares of green space per 1,000 people [20]. Highland had 65 GP practices, with 29 having access to a Community Link Worker involved in social prescribing. The GHPr was embedded within this general social prescribing service and the Active Health Project, an online social prescribing programme aimed at physical activity. During the evaluation timeline, a Green Health link worker was also employed by the National Park Authority, with dedicated funding, to deliver a structured GHPr pathway.

### North Ayrshire

North Ayrshire is comprised of rural and urban, island and mainland communities, inhabited by approximately 134,000 people, with 43% of urban areas comprised of publicly accessible greenspace and 36 hectares of green space per 1,000 people [20]. North Ayrshire utilised two methods of embedding GHPrs in existing pathways: a formal physical activity referral pathway called ‘Active North Ayrshire’, coordinated by KA Leisure; and social prescribing, which included multiple health and social care pathways e.g., via a community link worker, GP, pharmacist, or physiotherapist.

### Data collection

#### Funding and resource data

Information on funding and resourcing for the GHPr in each area was sought from the programme managers and officers. An Excel sheet was developed with questions relating to top-level programme budget, budget split and staff make-up, and several questions breaking down how staff time and resources were utilised. Completeness and nature (e.g. more descriptive than numeric) of the data was limited due to the fractional nature of the staff roles and the complexities of the programme funding. The aim was to get as much information as possible from each area.

#### Interview participants

The main data collection approach was via interviews with referrers, link workers, activity providers, and service users across the three areas. To achieve maximum variation in the views and experiences of the sample, we aimed to interview up to 18 people across three coordinator stakeholder groups (referrers, link workers, activity providers), with two people per area per stakeholder group (e.g., two referrers from Dundee). Additionally, we sought interviews with up to five service users from each area. For both coordinator and service user groups, an online advert explaining the study with a link to register interest was circulated through members of the project steering group and the three local Green Health Partnership steering groups, with a particular focus on cascading through green health activity providers to engage service users. Upon accessing the link, participants were presented with the information sheet and consent form, and following consent, were asked to provide demographics details (see Table 2) and contact details to arrange an interview.

Five variations of the interview schedule were developed to explore APEASE domains. One version each for referrers, link workers, and providers, and two versions for service users (one for those who participated and one for those who declined the referral). Interview schedules included the following topics: role in the GHPr and how it works in their area (coordinators only); referral process, invitations to participate, and awareness of the programme; journey from referral to green health activities; experiences of attending or coordinating green health activities; any additional barriers and facilitators; and anything that interviewees would like to change about GHPrs. The schedule for service users who declined the referral focused on the initial referral process, reasons for declining, and any additional barriers and facilitators. A copy of the service user interview schedule for those that engaged in the GHPr is provided in Supplementary File 1.

### Data analysis

#### Funding and resource data

Responses to questions asked in the spreadsheets were summarised, particularly highlighting reported differences between areas. More detailed quantitative information in Dundee allowed an estimate of the time and associated cost to the service of delivering an individual referral: Dundee responders outlined the activities involved in the referral pathway, and the typical duration each step takes. These times were multiplied by suitable hourly rates (‘unit costs’ – taken from Jones et al. 2024) to estimate the cost to the service of delivering a referral. Costs are reported in 2022/2023 values and the analysis assumes an hourly rate based on a ‘Support and Outreach Worker’, annual salary £24,900 (including oncosts), based on national figures [£19.73 and £31.39 per hour when considering respectively i) salary and oncosts and ii) salary, oncosts, direct, indirect and capital overheads – see [21] for details].

#### Interview data

Audio recordings of interviews and focus groups were transcribed by a General Data Protection Regulation compliant transcription service to produce verbatim transcripts. The analysis aimed to explore how Acceptable, Practicable, Effective, Affordable, and Equitable GHPrs are, and whether there were any Spillover effects (APEASE criteria; [17–18]). Definitions of each theme, alongside a project-specific example, are provided in Table 1.

**Table 1:** APEASE criteria definitions

Coding Theme	Definition	Project specific example
Acceptability	How far is what is proposed acceptable to important stakeholders, e.g., the target group, those delivering the intervention, funders?	Do staff and service users think the GHPr programme is acceptable? For example, attitudes or feelings about the programme.
Practicability	How far is what is proposed able to be implemented at the required scale, with the required quality for as long as will be required?	Do staff and service users think the GHPr programme is practical? For example, how easy it is for service users to get involved in the GHPr.
Effectiveness	How far will what is proposed achieve the policy objectives and provide value for money?	Does the GHPr programme improve health and wellbeing outcomes for service users?
Affordability	How far can what is proposed be achieved within an available budget?	Is the GHPr programme affordable for service users? For example, getting there and engaging.
Spillover effects	What effects, good or bad, will what is proposed have beyond the target behaviour?	Are there unintended outcomes, whether good or bad, for GHPr service users?
Equity	What impact will what is proposed have on health and social inequities?	Does the GHPr programme benefit (or not) certain groups of service users over others?

Analysis of the transcripts included both deductive (e.g., codebook) and inductive (reflexive) thematic analysis approaches [22], and utilised both NVivo software (version 12, QSR International Pty Ltd, Melbourne, Australia) and Microsoft Excel. The following step-by-step process was followed:

1. A ‘node’ was created for each of the six domains of the APEASE criteria.
2. Using a codebook thematic analysis approach, any relevant passage related to any of the six APEASE domains was coded to the relevant node/s.
3. All data attached to each node representing an APEASE domain were reviewed, and if the data was a clear representation of a domain, the quote was extracted to an Excel sheet alongside the participant identifier, stakeholder group, and reasoning for why this quote was clearly linked to this domain. There was one datasheet per APEASE domain.
4. All data extracted to the Excel sheet, within each APEASE domain were then analysed with a second layer of reflexive thematic coding [22]. Preliminary code names were assigned to each data item and iteratively developed throughout the coding process, led by NH, and supported by IF, LL, and NL (all experienced qualitative researchers).
5. From these code names (and related data), initial sub-themes were generated that encapsulated multiple code names, and represented the breadth and depth of the data, by featuring quotes from multiple interviews, and often across stakeholder groups (e.g., both referrers and activity providers in different areas). A second member of the team then reviewed the sub-themes to enhance the richness and nuance of coding, rather than seeking consensus [22].
6. Sub-themes and supporting quotes were then discussed with the project and advisory groups, and public involvement members to sense-check and refine final interpretations.

### Patient and public involvement and engagement

Patient and public involvement and engagement was embedded through both the involvement of three members of the PHIRST Connect Public Involvement in Research group (PIRg) and three lay public contributors recruited to sit on the project-specific advisory group. Two members of the PIRg supported project development since inception, attending project meetings, commenting on the protocol, and one member (SH) was involved in developing the evaluation approach, interview schedules, supporting data analysis, and co-authoring this manuscript. The chair of the PIRg led on involving public members and attended advisory and project steering groups.

## Results

### Funding and resourcing

Programme managers and officers provided information on GHPr budgets, resourcing and other contextual information. Programme staff could not always isolate detail specific to the referral pathway as opposed to information about the wider Green Health Partnerships. The number and type of staff delivering the pathway varied widely, with some tailoring of staff and structures related to the different geographies e.g., Highland had ‘local link workers’ to address the very large geographic area it supports. Often, staff delivering the GHPr had responsibilities beyond the pathway. For example, the Senior Project Officer in North Ayrshire developed, supported, and promoted the pathway and was allocated 1.5-2 days a week for this. Green health activities were typically led or supported by professionals from a range of backgrounds (e.g., link workers, active officers, rangers) or volunteers, who received no formal reimbursement aside from some expenses.

Green health activities offered included: walking; cycling; growing, gardening and environmental conservation; nature arts and crafts; outdoor learning; citizen science; outdoor volunteering; outdoor sports; relaxation and mindfulness; gentle movement; and art groups. In various ways, all three areas noted that there was only very limited routine data collected. For example, one region noted only collecting information on whether a service user received a referral but could not provide detail on whether service users went on to take-up a suggested green health activity. This reflected challenges elsewhere in the evaluation around the lack of routine monitoring of service user demographics, attendance levels, and related health and wellbeing outcomes. This makes it hard to quantitatively assess the impact of the GHPr and monitor equity of access.

### Green health prescription (GHPr) in Dundee

More detailed information was available from Dundee, which allowed a granular breakdown of GHPr costs and resources. Their £100,000 budget per annum was split £54,000/£46,000 between staff and non-staff costs. Their staff consisted of a Coordinator, Project Worker, and Senior Project Worker. However, sometime after set-up, the Project worker left, and the referral work was split between the other two roles.

**Fig. 1:**
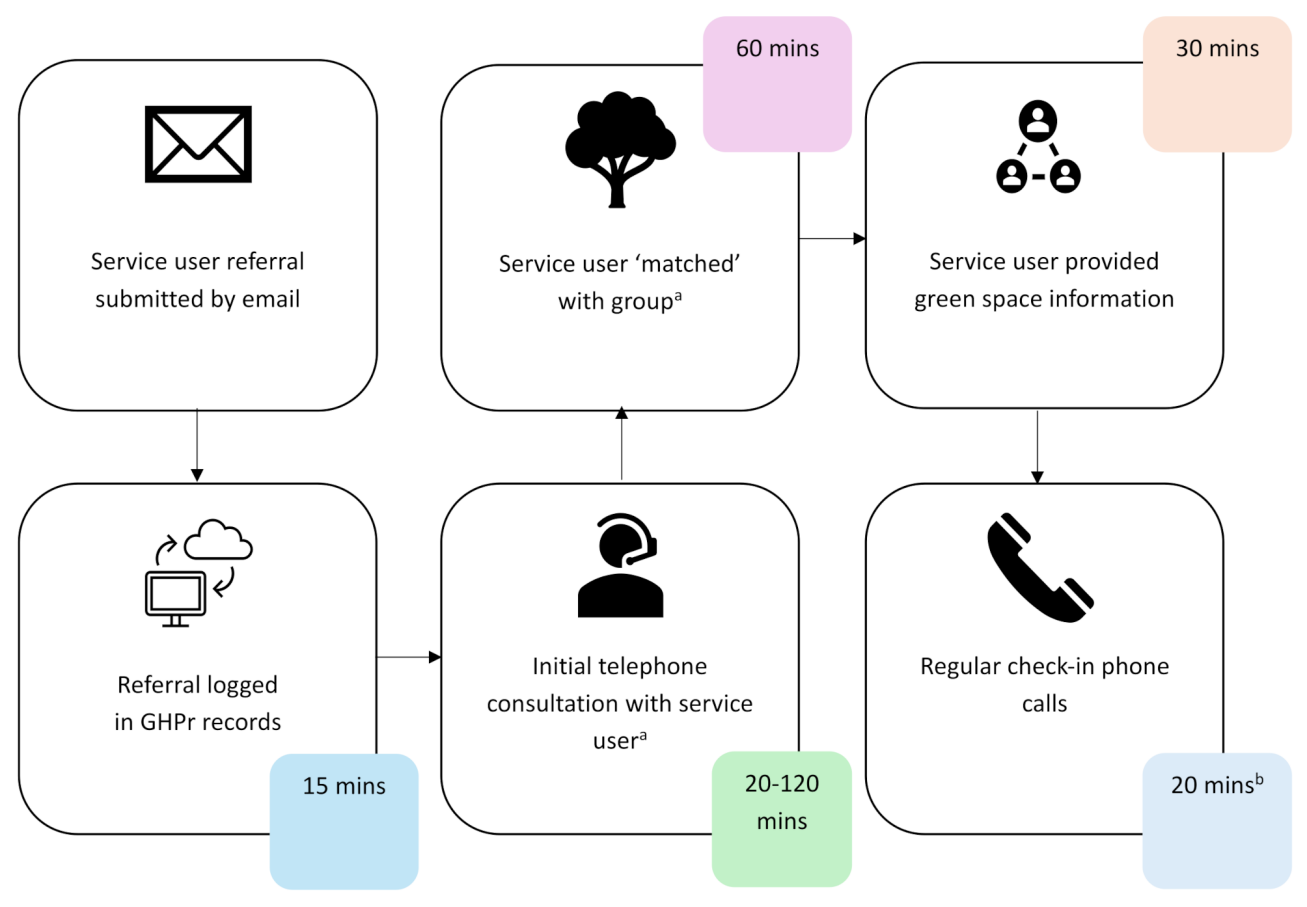
Time taken for each step of the Dundee GHPr ^a^Service users are ‘matched’ with a green health activity, taking into account information such as location, disabilities, etc ^b^Regular check-in phone calls are around every 2–3 weeks. No information was provided on how long these check-in phone calls last. In the costings here, we have assumed one check-in phone call

Figure 1 shows the standard steps of a GHPr in Dundee, with the typical time taken to deliver each. Typically, it takes a total of 195 min, ranging from 145 to 245 min, with the time range varying with the length of the initial telephone consultation (typically 70 min). Assuming a Project Worker delivers all parts of the referral, we estimate it to cost the GHPr approximately £64 per referral (£48-£81) when just considering salary and oncosts, or £102 per referral (£76-£129) when wider costs are also considered. This does not account for the resources/costs of logging a referral with the service (e.g. time with GP for initial signposting/referral).

### Interviews

A total of 28 one-to-one interviews were conducted between April and October 2023. This covered four referrers, seven link workers, five activity providers, and 12 community members. The breakdown of stakeholder group roles, and overall coordinator and community member demographics are provided in Table 2. Findings are presented below under each of the APEASE criteria headings.

**Table 2:** Interviewee participant demographics (*N* = 28)

Stakeholder group	Stakeholder roles	Age (years): Mean (range)	Gender: *n* female (% Female)^a^	Ethnicity: *n* (%)	Long-term condition or disability: *n* (%)
Referrers (*n* = 4)	GP, Pharmacist, Physiotherapy lead	45 (37–52)	2 (50%)	4 (100%) White British	0
Link workers (*n* = 7)	Green Health Link worker, community link worker, Active Health Link worker, GHP Officer	37 (24–49)	5 (71%)	7 (100%) White British	0
Activity provider (*n* = 5)	Countryside Ranger, Health & Wellbeing Manager, Green Health Prescription activity lead	49 (40–56)	5 (100%)	5 (100%) White British	0
Community members (*n* = 12)		49 (27–75)	4 (33%)	7 (58%) White British	4 (33%)

^a^ Participants were asked, online, about the gender they identify with, with the following options available: Female; Male; Other (please specify); Rather not say

### Acceptability

#### Positive perception of the programme and/or green space

Both the concept of the GHPr and green space in general were viewed positively, with service users feeling grateful for opportunities to engage with activities, and reports from both staff and service users that prescriptions had been beneficial.

*“I think[…]*,* because we have such an amazing kind of natural resource all around us*,* […] fairly accessible for most people*,* you know*,* I do think it is something that works really well and I think*,* you know*,* most of the people we’re talking to do kind of appreciate that.”* (Link worker).

#### Staff characteristics and communication skills

Activity providers were often viewed as skilled communicators, who were helpful, engaging and approachable. Link workers who had knowledge of local activity options, helped to guide service users through the programme and recommended activities aligned to service user interests. Service users sometimes preferred that the GHPr was introduced to them by someone they knew from their community, as well as more diverse and representative activity providers. However, GPs who championed the GHPr approach were able to reassure service users of its legitimacy.

*“I always take on more healthcare advice from my GP because whenever I get prescriptions*,* I always ask my GP if it’s safe*,* if he has more available information*,* so how effective this form of treatment*,* for me to*,* it was more like my GP was very close to me and really helpful for most of the decisions I took. Even down to the point of opting for a Green Health Prescription*,* that has really helped my social wellbeing.”* (Service user).

#### Intrinsic and emotional influences

Service user participation in green health activities could be inhibited by feelings of isolation. Similarly, anxiety, either in general or related to a lack of familiarity with activities and how to access them, may also prevent uptake. Potential service users may have low levels of motivation to change, a lack of interest in health and fitness, or a perception that activities are for “health freaks only” (Service user). This may result in those who are more in need of additional support participating less. One service user suggested offering introductory sessions so potential users can “put their toe in the water”. However, for some service users, acceptability may grow naturally over time as they attend sessions and become more “comfortable”.

*“I think another thing that could be improved*,* or what I’ve seen is challenging is that often participants do not have the means in place to be able to actually attend the activities. So*,* they might feel too isolated*,* they might be too anxious*,* they might be uncertain about how to do it or how to get there and I have seen that repetitively with participants.”* (Green Health project worker).

#### Providing an alternative treatment and pathway

Some staff welcomed the addition of a non-medication-based option, but some service users viewed GHPrs as inferior and possibly inequitable, with one perceiving that the referrer was trying to avoid helping them by referring them to “another process entirely”. Some staff perceived the GHPr as more sustainable as it represented an open-ended offer, preventing service users from ‘falling through the net’ of alternatives options such as a time-limited exercise referral programme. Referrers also felt they benefited from having a link worker in place to keep track of referrals and simplify the referral process. Others did not feel the GHPr was fit for purpose yet, with service users getting lost in the system and existing exercise referral pathways being used instead.

*“I guess I tried to do green prescriptions before we had a link worker. My issue there was always the keeping track of everything that was available. Things were always updating*,* you know*,* and maybe I was sort of getting behind as well. So now*,* these days*,* we’ve got a community link worker*,* so I tend to think about her and refer people on to her*,* which is quite useful*,* it really helps and I tend to get feedback from her which I find very beneficial. It’s good.”* (Referrer).

#### Characteristics and variety of activities

Variety in activities seemed key for service users, with increased local availability and range of activities desirable. It was important for service users to feel at ease in the locations where they were engaging with green health activities. Service users also appreciated having providers to talk to who were passionate about nature. Some enjoyed activities that gave back to the community (e.g. community gardening), prompted specific feelings (e.g. calm), or provided opportunities to be mindful (e.g. time to think). Activities offering social connection were perceived positively, as was variety in intensities of exercise for different needs. Tailoring to service users’ needs and interests, for example, offering a range of options rather than a single activity, was also recommended.

*“It shouldn’t be like*,* okay*,* this is what you’re going through*,* this is what you have to do*,* it should be like this is what you’re going through*,* these are a couple of things you could do that would make you feel better*,* and then work with you to see which of them fits you and then which of them best would help you get better.”* (Service user).

### Practicability

#### Awareness and knowledge of service

A lack of awareness of GHPrs as a referral option was noted across areas, with some participants feeling that members of the public may not be aware programmes like GHPr existed. In some cases, this led to referrals to exercise schemes where a GHPr may have been more suitable. Awareness about GHPrs could be supported in two ways, firstly with improved communication to referrers from the wider Green Health Partnership work; secondly through a range of public facing messaging, such as social media, to inform potential service users about GHPrs. Interviewees also noted that GPs and link workers (some of whom were not employed by GHPrs) sometimes lacked knowledge about GHPrs, which could act as a barrier to service user engagement.

*“So maybe*,* again*,* strengthening that communication between Green Health and the NHS*,* for example. Or I think also utilising social media platforms a bit more*,* making sure that is always updated and that is transparent as well. Yeah*,* just ensuring that consistent communication is made across all platforms and across all sectors. That all*,* yeah*,* referrers are aware of everything consistently and also really know what Green Health is about.”* (Green Health project worker).

#### Communication format and style

In addition to previously noted communication styles under the theme of Acceptability, effective communication between service users and professionals was key for service user engagement. Having the opportunity to ask questions and get sufficient information made service users feel confident about engaging in green health activities. In some situations, communication about what was on offer could have been better. Remote or digital communication was also identified as a barrier to service users without access to the internet.

*“Well*,* possibly better communication because I didn’t get a referral through my GP*,* it was just*,* just by chance that I saw the information*,* so I think communications and maybe for some people that English is not their first language*,* that might be a barrier.”* (Service user).

#### Location of activities and transport

Location and transport infrastructure was often challenging for service user engagement. Some GHPr activities were in areas that made access for participants difficult due to limited public transport options and poor transport infrastructure (regular bus timings), especially hindering those with mobility issues. A wider range of activities in locations with adequate transport options may make green health activities more accessible.

*“Is there local transport links that will get you to that Green Health activity*,* you know*,* that would fit with the timings... There’s not trains that go everywhere and buses*,* bus routes can be a bit strange sometimes actually... I’ve learnt that they don’t always directly link up all the times*,* you have to sort of change bus*,* you know*,* to get between different towns.”* (Link worker).

#### Supporting initial service user engagement

Attending green health activities may be daunting for service users experiencing anxiety, lack of confidence or other forms of mental ill health. Support from activity providers or link workers when attending activities for the first time was suggested to improve engagement. In addition, a buddy system with already existing service users accompanying newcomers was suggested as potentially beneficial.

*“... And sometimes maybe that would make a difference is if there was more capacity for people that were really struggling to or just taking that first step along to a new activity*,* to a group*,* can be really hard for some people. So having somebody to chum them along to that....”* (Link worker).

#### Referral processes and systems

In Dundee, a dedicated referral pathway (see Fig. 1) is in place due to agreements made between referrers and link workers, including the ability to add to medical records and collect routine service user data. Where referral processes were disjointed, this was seen as a barrier to GPs engaging with the GHPr, with the perception that pre-existing exercise referral programmes are more convenient for referrers. Furthermore, lack of monitoring of patient feedback, and IT systems for green health referrals may lead to poor communication between referrers and link workers.

*“... It doesn’t stamp in the notes very well that we have referred a patient. Although I can do it through a computerised system*,* if the next GP comes along*,* when they look at the notes*,* they won’t know it’s happened... with the feedback*,* there’s no way to know that the feedback has happened unless you enter the certain part of Elemental*,* which is in the notes. So*,* the GP won’t see all the pretty information sometimes that’s been chatted about…it’s like a completely separate service unless we go looking for it.”* (Referrer).

### Effectiveness

#### Support for mental health

It was reported that service users experienced reductions in depression and anxiety, and those that had used the GHPr in conjunction with or instead of mental health-related medication found it beneficial in reducing symptoms.

*“... my psychiatrist really didn’t want me to rely more on sedatives as a form of recovery for my mental health because they don’t really have a lasting effect*,* well I think therapy was the better option*,* but I think the best is Green Health*,* Green Health because the combination of Green Health and therapy sessions really helped with me.”* (Service user).

#### Social connections

Service users and staff reported that those engaging in green health activities increased their socialisation and connection with others, and experienced associated benefits e.g., sense of community, learning from others.

*“When I join walks*,* people tell me stories of how they used to be lonely and not able to go for a walk like they do now and the amount of change that has come out of them just because of joining that group.”* (Link worker).

#### Emotional benefits

Service users and staff reported emotional benefits of taking part in GHPrs, including improvements in mood, confidence, feeling less angry, a sense of calm, managing thoughts, self-empowerment, mindfulness, focus, and “zest for life”.

*“You can’t help but feel better when you look at anything in nature. Doesn’t have to be trees*,* I mean*,* I particularly like trees but*,* you know*,* bumblebees*,* insects*,* just*,* it doesn’t matter*,* leafs. It just makes you feel… and it’s so calming*,* I just feel so calm. The woodland bathing is just so calming. You just sit there and listen*,* you know.”* (Service user).

#### Physical benefits

Service users reported changes in health behaviours such as healthier food substitutions and increased physical activity. This in turn led to the perception that a range of positive health outcomes were experienced including increased stamina and strength, sleep quality, and ability to control weight.

*“I feel more able to*,* I’ve got more strength and stamina for the grandchildren and also within my caring role I can do things that perhaps someone of my age group if they didn’t do these things might not be able to carry out.”* (Service user).

### Affordability

#### Importance of free access for engagement

Staff highlighted the potential for cost to be a barrier to engagement with green health activities. Activity providers emphasised the importance of activities being low-cost or free at the point of access.

*“Yeah*,* so it’s entirely free for service users and it would be very important to me that it remained so. Yeah*,* we’d only ever do it if it was fully-funded*,* yeah.”* (Activity provider).

Reassuringly, service users indicated that green health activities were affordable, highlighting the minimal attendance fees and efforts made by service providers to make activities accessible and affordable. Examples included, where possible, providing free refreshments and transport to enable engagement, and other efforts to reduce cost concerns such as accessing additional funding for activities to allow them to remain free or low-cost.

#### Need for specialist equipment

Even where attendance was free or low-cost, some green health activities required specific equipment or clothing to fully participate (e.g., waterproof clothing). In these cases, service providers were able to reduce barriers as much as possible by keeping requirements to a minimum or providing equipment and clothing where required.

*“I tried to make sure there’s no barriers in terms of the clothing and things that people have to wear so I usually have a small supply of like waterproof jackets and trousers and boots and things like that that I can supply people*,* so they’re not really expected to come out there like dressed for climbing up a mountain or anything*,* they’re just sensible outdoor stuff.”* (Activity provider).

#### Travel costs

Service users highlighted travel related costs as a key potential barrier to accessing and ongoing engagement with green health activities.

*“Initially it [cost of travel] was like a challenge*,* but I began to enjoy the whole [green health activity] process*,* so it was more like it was really cost-effective and it was worth it.”* (Service user).

Coordinating staff commented that there may be a need for additional financial support to cover the cost of travel for some service users to help encourage and maintain engagement.

### Spillover effects

#### Belonging through shared experiences and new friendships

Meeting with ‘like-minded’ people that were living with similar experiences or conditions was seen as a positive. Participating in green health activities also provided a sense of belonging, sometimes connected to the local community. This could provide opportunities for friendships that could flourish outside of the sessions, helping to combat loneliness.

*“... I think that opportunity and watching how these people suddenly bonded and became good friends and were then taking it further to meet up in their own time and stuff like that*,* that’s how positive giving somebody an opportunity in a green space just to crack on and do what they want to do has for people.”* (Activity provider).

#### New skills and experiences

Gaining new skills through activities such as planting and gardening helped service users with addiction recovery, confidence, and feelings of success. New experiences (e.g., leading a green health activity) were perceived to break down barriers to engagement with activities and led to feelings of increased self-esteem.

*“... another thing which I’d never really kind of known much about was kind of like collecting things*,* you know*,* like foraging for things that just grow naturally kind of all around you*,* like things like dandelion*,* you know*,* we did that*,* and we made dandelion cake*,* like the wild garlic*,* collecting that as well... So that was interesting as well*,* kind of learning all these new things.”* (Service user).

#### Openness with others

Participating in green health activities opened service users up to new social experiences and getting connected with new people. This could be in the form of being more tolerant and listening to others in the group, which in turn made them more willing to share experiences and gain an understanding of other people.

*“... it helps you to*,* you know*,* to also share with people*,* I think that’s one of the important things*,* you are able to build trust in people and that way sometimes you even don’t know when you start sharing*,* and the process of sharing you begin to get other people’s perspective*,* and you know*,* your perspective is also well improved*,* and I think and overall it’s more like a peer support kind of thing*,* that happens.”* (Service user).

### Equity

#### Inaccessible activities for mobility and disability

Some activities by their nature (e.g., accessing wooded areas) are not well suited to wheelchair users or those with poor mobility. Some suggested a more tailored approach to green health activities, to support those with differing physical abilities. For example, creating new paths to accommodate for uneven ground and providing disability parking facilities. Others felt that, where possible, accommodations were made (e.g., seated options). However, sometimes these accommodations were online or home-based options, rather than activities in green spaces.

*“So obviously it wouldn’t have been ideal for somebody that was in a wheelchair or used a walker or whatever*,* but I know she has had people attending the course*,* one woman who’s using a mobility scooter and another elderly lady who has a walker*,* and it was just fewer activities. There’s just more done in the garden rather than going into the actual forest.”* (Service user).

#### Cultural barriers

There were several mentions of activities possibly not being attractive to different cultural groups or men. It was noted that within some cultures, the topic of mental health was not as socially accepted, which may act as a barrier to engagement for certain groups. Additionally, some GHPr activities may be perceived as more suited to female participants, raising anxieties for some men about how they may be perceived during engagement with nature-based activities.

*“... the culture that they come from mental health is a complete taboo so you can’t*,* you know*,* you can’t talk about it*,* they would never allow themselves to be referred onto something because that would be an identifier that they were experiencing poor mental health which would be just completely unacceptable in their culture and their families and things.”* (Activity provider).

#### Minimising travel difficulties

Travel issues were often cited as a potential problem, whether the need for a car, or issues with public transport (e.g., social anxiety, mobility challenges). However, there were examples of activity providers collecting service users from bus stops, and even one activity providing transport from people’s homes.

*“I think there’s a few who might struggle to actually come to us because they don’t*,* again they don’t really have that confidence to actually jump on a bus*,* they don’t know where to get off. I have had to go hunting for people before [laughs] because*,* and the bus stop’s just right beside us*,* but they’ve got off earlier and stuff like that.”* (Activity provider).

#### Flexible options for those caring or working

There were comments around the (un)availability of sessions during evenings and weekends for those with working and/or caring responsibilities. Activities were, however, able to engage parents with young children.

*“I suppose the other barrier is those who work*,* there are not an awful lot of activities that are available in an evening and in a weekend so I have had patients before that want to go out and do things*,* but it might be like ad-hoc activities that they end up engaging in.”* (Link worker).

## Discussion

Based on the available data on costing and resources, it was difficult at times to clearly differentiate between wider Green Health Partnership information and details specific to the GHPrs. When more granular information was available, an estimate of the time taken for a service user to progress through referral stages was possible. This evaluation also provided detailed feedback from staff and service user perceptions and experiences of the GHPr. Staff and service users generally found the concept of using green spaces and the GHPr acceptable, and there were reported improvements in a wide range of physical and mental health, and social outcomes for service users who engaged. The GHPr was also considered affordable in terms of the limited (if any) cost to attendees of the green health activity sessions. There were some barriers to engagement with green health activities for service users such as travel cost and location, and for those with mobility issues with some activities having limited accessibility. Although service users perceived referrers, link workers, and activity providers to be good communicators, there were times where awareness of the GHPr and knowledge about the finer details of what was on offer were lacking. Relatedly, the main barrier for staff, particularly those referring people into the programme, was the lack of strong underpinning IT infrastructure in terms of noting that a referral had been made, communication with link workers, and feedback and data capture to reflect on service user access and progress.

### Comparison between areas

Alongside the difficulties in highlighting funding and resource data specific to the GHPr in some areas, there were other notable differences. Staff in Highland and North Ayrshire often had responsibilities beyond the GHPr e.g., the Senior Project Officer in North Ayrshire was allocated up to 2 days a week to support GHPr. There was also some tailoring of staff and support structures related to the different geographical areas e.g., Highland had local link workers to address the very large area it supports. Similarities across areas included the green health activities offered (e.g., walking; cycling; gardening and environmental conservation; nature arts and crafts; outdoor learning), the wide range of professionals and volunteers supporting the GHPr, and challenges in collecting routine data.

### Comparison with other studies

Findings from interviews with service users, and the perspectives of staff regarding service user experiences, showed that there were high levels of acceptability, and a range of positive outcomes reported. This is consistent with evidence from service users with mental health needs who reported improved wellbeing from green social prescribing [23], and those with long-term conditions from other social prescribing programmes that improved confidence, health behaviours, and self-management of conditions, and reduced social isolation [24]. Furthermore, reported benefits from service users align with previous research relating experiences in green space with mental health (e.g., [2, 25]), and better social [7] and physical health outcomes [4]. Lack of awareness of and knowledge about GHPrs reported in the current study links back to earlier work with a range of GHPr stakeholders who identified the need to have a “one-stop shop” for accessible information for service-users and staff [11].

It was clear from the findings of this study that there is an urgent need for improvement in data linkage within IT systems, and routine monitoring data, in line with a national evaluation of green social prescribing programmes in England [23]. The requirement for robust evaluation to evidence outcomes was identified as a key need in early research with health professionals involved in the strategic planning of GHPrs [10] and remains a key ongoing challenge. The current findings also align with the guidance generated from a recent expert group of practitioners, researchers, and policymakers exploring nature-based social prescribing programmes (NBSP; [26]). Two major themes focused on the need for capacity building of staff to deliver NBSP, and for standardisation of implementation and evaluation across areas and contexts [26]. Any standardisation of implementation would have to be balanced with the unique context of each area, which often operate with different workforce capacity and structure, funding streams, existing social prescribing pathways, levels of deprivation, and health challenges for their population. Drawing on the Consolidated Framework for Implementation Research [27, 28], standardisation of the GHPr could be supported by ascertaining what counts as ‘‘core components’ (the essential and indispensable elements of the intervention)’ (e.g. access to a choice of green health activities) and what counts as an ‘adaptable periphery’ (adaptable elements, structures, and systems related to the intervention and organization into which it is being implemented)’ (e.g. the professional role of the referrer). In theory, standardisation of evaluation should be more straightforward by agreeing a Common Outcomes Framework (e.g., [29]). In reality, even when very light touch quantitative evaluation (i.e. with a four-item wellbeing questionnaire) has been attempted, it has still been challenging to ensure consistent data collection across areas [23].

### Implications for policy and practice

Policymakers should be keen to continue funding and supporting green health activities that are accessible, affordable, and provide benefit. Without sustainable funding there is a risk of losing the momentum, knowledge, and capacity that has already been built by GHPrs. Furthermore, making data collection and linking a more integral part of future referral pathways would benefit every group of stakeholders from service users through to those commissioning GHPrs. From a service user perspective, having their progress monitored would help recognise any improvements they have made, and provide a more active safety net if they were still struggling, helping with continuity of care and potentially prompting additional treatment that might be required. From the perspective of frontline staff, being granted adequate workload, resource, and training to be able to help monitor service users and become a partner in evaluating the programme would help evidence the impact of their hard work, improve job satisfaction, and provide employability skills transferable to a range of future or other roles. From a referrer perspective, they would be more likely to refer into a GHPr if they could monitor service user progress through an embedded feedback mechanism. From a policy maker or commissioner perspective, good quality data collection and additional resource for GHP officers should be a requirement of funding any future GHPrs. Without this improved data capture, GHPrs will not be able to effectively evidence service user outcomes, to guide future policy and funding decisions. Subsequently there is real danger of not meeting key strategic aims of government and NHS Scotland, such as valuing green space and increased provision of green health activities for health (e.g., [30]).

### Implications for research

Researchers need to more consistently partner with GHPrs (and other social prescribing) programmes to work on embedding evaluation. Designs like RCTs are rarely feasible (or appropriate) to evaluate complex real-world public health programmes [13], so there is a need for creative and novel approaches that might ease the burden on the staff and volunteers involved. One possible option could be a quasi-experimental design whereby areas with GHPrs are matched with areas that do not operate the scheme, on factors such as population level, deprivation levels, and amount of green space available (see [31] for an example of area matching). There are also new observational methods of data collection that do not place addition burden on the workforce that could be implemented. For example, the Method for Observing pHysical Activity and Wellbeing (MOHAWk; [32]) offers a novel way of capturing validated data on sedentary behaviour, physical activity, and wellbeing in naturalistic settings, that is collected by researchers.

A related challenge with these types of evaluation is the recruitment of service users who have rejected a referral or accepted the referral but dropped out before getting to the first activity session. While this evaluation managed to speak to one service user who did not accept a referral, this remains an ongoing challenge for research and evaluation. If routine data monitoring and evaluation were embedded in pathways – such as the GHPr – there may be opportunities to engage with people who decline referrals, to ask if they can be contacted to discuss their decision to decline. Research often misses out on important data around issues of acceptability, affordability, and equity by not managing to speak to more people who choose not to engage with such programmes.

### Strengths and limitations

A strength of this evaluation was the analysis method which applied the APEASE criteria as an overarching framework but still allowed for more reflexive sub-themes to be generated. This is the first study, to the authors’ knowledge, which has combined deductive coding using the APEASE criteria with inductive sub-themes, allowing for richer data analysis. It should, however, be noted that starting from a deductive framework may have restricted the range of reflexive sub-themes that could be generated as they still had to fit within an existing coding structure. A further limitation was the extent to which it was possible to recruit service users who had experienced all three stages of a formal pathway i.e., referral, discussion with a link worker or GHP Officer, and attendance at a green health activity session.

The nature of the GHPr in some areas was informal, and therefore, the service users interviewed experienced different combinations of elements (e.g., full pathway, referral straight to a green health activity, or just a green health activity via self-referral). This speaks to wider challenges with evaluating community-based approaches, as they are often not branded or recognisable by name (see example of recruitment challenges for service users in [33]). Social prescribing programmes can be ad hoc in nature, without standardisation or existing data capture systems associated with more well-established referral pathways, and there is a need for better evidence generation [34]. A related limitation is that this evaluation was conducted remotely, which produced challenges in accessing service users directly (i.e., there was a reliance on recruitment via service staff). Furthermore, the collection of data was limited by the lack of clear distinction in Highland and North Ayrshire between funding and resources that were allocated to wider Green Health Partnership activities versus dedicated to the GHPr. This reflected that GHPrs in these two areas were embedded into existing social prescribing approaches rather than being a standalone pathway with dedicated funding.

## Conclusion

This evaluation of three GHPrs across Scotland showed that when service users engaged in green health activities, they reported a range of benefits, and appreciated the support offered from the staff and volunteers. The findings highlighted areas where the GHPrs could provide a better experience for service users and staff. The GHPrs have not been well incorporated into existing referral and data systems, and this presented barriers for referrers and coordinators to fully engage in the programme, and for providing a sustainable programme offer across the three areas in Scotland. There is a strong need for improvements in data capture and evaluation as a routine element of the GHPrs so that staff can track service user progress and those funding the programmes going forward can make strategic decisions based on more robust evidence.

## Electronic supplementary material

Below is the link to the electronic supplementary material.

Supplementary Material 1

## Data Availability

The participants of this study did not provide written consent for their data to be shared publicly, and the data contain some information that could compromise the privacy of research participants; therefore, supporting data are not publicly available. However, interview schedules, and data supporting the findings of this study are available from the corresponding author upon reasonable request.

## Abbreviations

APEASE: Acceptability, Practicability, Effectiveness, Affordability, Spillover effects, Equity
GHPRr: Green Health Prescriptions
GP: General Practitioner
NBSP: Nature-based social prescribing programmes
NHS: National Health Service
PIRg: Public Involvement in Research group
RCT: Randomised controlled trial
