# Supplementary material for: An evaluation of Scottish green health prescriptions using the APEASE criteria
Source: BMC Prim Care. 2025 Feb 22;26:50. doi: 10.1186/s12875-025-02746-9 (PMC11846349; doi:10.1186/s12875-025-02746-9)
Supplement: Supplementary file 1 — Supplementary Material 1 [file 12875_2025_2746_MOESM1_ESM.docx]

**Supplementary File 1 - Indicative Interview Schedule for Service users**

The researcher will introduce the overall aim of the research and the goal of the interview. It will be explained that the aim/goal is to explore Green Health prescriptions (GHPr) within Scotland. This includes looking at:

- How GHPrs work within (Dundee/Ayrshire/ North Highland)
  - Green Health prescriptions are a form of social prescription from either a GP, Physiotherapist, or other health care professional to take part in physical or social activities that happen outdoors. The aim of these prescriptions is to support people’s health and wellbeing. This might include being referred from a GP to a Green Health Link worker, or community link worker where they will talk about different outdoor activities/ groups within the local area to see what a patient will be most interested in.
- Who they work for
- What settings they work best in
- Any barriers or facilitators to engaging with GHPrs.

**When designing the interview schedule, questions were mapped to APEASE elements with the following: Acceptability = Ac; Practicability = P; Effectiveness = Eff; Affordability = Af; Spillover effects = S; Equity = Equ.*

**Referral/invitation/awareness of GHPr**

- **As part of our evaluation, we would like to ask you questions around green health activities, these are activities which take place outdoors and allow you to spend more time in nature. They might include, group forest walks, forest bathing, Nordic walking, community gardening and so on.**
- **Having taken part in a Green Health Activity, would you mind telling me how did you first find out about Green health activities?**
  - Possible prompts: referral/word of mouth/ signpost/ community link/ GP practice
- **(if referred) Can you talk me through your experience of being referred to a Green Health Activity within (Dundee/ North Ayrshire/ Highland)? (Ac/P)**
  - Possible prompts: who referred you? Did you accept? If not, what made you say no? What was the waiting time like? Was it clear what you were being referred to and why? What were you referred for e.g. improvement in mental health/health condition/improving physical activity…
  - What went well, what did not go so well and why?
- **Can you tell me what other programmes/ activities (if any) you have been referred to/ involved in to help support you, apart from the Green Health activity?**
  - What were you being referred for? e.g. mental health/health condition/ improving PA.
  - How did this referral experience differ?

**The journey from referral to green health activities**

- **Can you tell me about your experience between getting referred and starting a green health prescription activity (Ac/P)**
  - Possible prompts: Talking to a link/community worker or GHP officer on the phone? Email contact or written information? Choice of activities and location? What would help this process work better?

**Experiences of GHPr**

- **Can you tell me about what Green Health activities you have taken part in? (Ac/P)**
  - Possible prompts: what were these like? How long did they last? How many sessions a week did you attend? Were the activities accessible from where you live? How many people attended with you? What were the providers like? What was good or bad about these activities? How could they have been improved the experience?
- **Have you experienced any challenges in attending or taking part in the Green health activities e.g., mobility issues or caring responsibilities**
  - Possible prompts: If so, what were these? Were you able to overcome them? Could you choose a different activity or could the service cater for your needs?
- **Through taking part in Green health activities, in what ways (if any) have you noticed a change or impact on your health and wellbeing? (Eff/S)**
  - Possible prompts: what were those changes? Where these impacts good or bad? What are the reasons for the impacts noticed.

If there are not impacts noticed, why do they think this happened?

- - You mentioned you were referred to X service beforehand, what was your experience like of this service? How was it different from GHPr activities?
  - Have you noticed any improvements in your condition from taking part in this service? How does it compare to any improvements from taking part in a GHPr?
- **Have you noticed any changes in your levels of physical activity as a result of your taking part in Green health activities? (Eff/S)** (May need to define physical activity – activity that makes you out of breath/sweaty)
  - Possible prompts: If so what has changed? When comparing to your activity levels before starting the programme?
- **Have you noticed a change in the amount of time that you spend in green spaces as a result of your Green health activties? (Eff/S)**
  - Possible prompts: if so, what has this change been? Why do you think this change has occurred?
- **Are there any costs involved with taking part in Green Health activities? (Af/Equ)**
  - Possible prompts: Travel costs? Costs of the activity? Parking? Equipment needed to take part in the activity.
  - Was this a challenge for you? Did any potential costs impact your decision in taking part in the GHPs?
- **Overall, can you tell us what it is like to take part in green health activities within (Dundee/North Ayrshire/ Highland)? (Ac/P)**
  - Prompt: what went well, what did not go well, and why?
  - Are there any improvements?

**Barriers and facilitators**

- **Can you think of any barriers that would stop people from engaging in Green health activities? (Equ)**
- **Can you think of any things that might encourage people to engage in Green Health activities?**

**Thinking about the future**

- **If you were in charge, what sort of changes would you like to see in how Green Health prescriptions be delivered? (Ac/P)**
  - Prompt: Improvements? Based on your experiences, what would work best? Other activities you’d like offered?
